# Plant-Produced Nanoparticles Based on Artificial Self-Assembling Peptide Bearing the Influenza M2e Epitope

**DOI:** 10.3390/plants12112228

**Published:** 2023-06-05

**Authors:** Elena A. Blokhina, Eugenia S. Mardanova, Anna A. Zykova, Liudmila A. Stepanova, Marina A. Shuklina, Liudmila M. Tsybalova, Nikolai V. Ravin

**Affiliations:** 1 Institute of Bioengineering, Research Center of Biotechnology of the Russian Academy of Sciences, 119071 Moscow, Russia; 2 Smorodintsev Research Institute of Influenza, Russian Ministry of Health, 197376 St. Petersburg, Russia

**Keywords:** influenza A, vaccine, plant, nanoparticle, M2e peptide, self-assembling peptide, transient expression, *Nicotiana benthamiana*, viral vector

## Abstract

Despite advances in vaccine development, influenza remains a persistent global health threat and the search for a broad-spectrum recombinant vaccine against influenza continues. The extracellular domain of the transmembrane protein M2 (M2e) of the influenza A virus is highly conserved and can be used to develop a universal vaccine. M2e is a poor immunogen by itself, but it becomes highly immunogenic when linked to an appropriate carrier. Here, we report the transient expression of a recombinant protein comprising four tandem copies of M2e fused to an artificial self-assembling peptide (SAP) in plants. The hybrid protein was efficiently expressed in *Nicotiana benthamiana* using the self-replicating potato virus X-based vector pEff. The protein was purified using metal affinity chromatography under denaturing conditions. The hybrid protein was capable of self-assembly in vitro into spherical particles 15–30 nm in size. The subcutaneous immunization of mice with M2e-carrying nanoparticles induced high levels of M2e-specific IgG antibodies in serum and mucosal secretions. Immunization provided mice with protection against a lethal influenza A virus challenge. SAP-based nanoparticles displaying M2e peptides can be further used to develop a recombinant “universal” vaccine against influenza A produced in plants.

## 1. Introduction

The influenza A virus is one of the most important respiratory pathogens affecting animals and humans. Vaccination represents the most effective way to prevent influenza disease. The influenza A virus evolves rapidly as a result of antigenic shift and antigenic drift [1]. Antigenic drift is caused by frequent point mutations during virus replication, affecting the antibody-binding sites in the hemagglutinin (HA), neuraminidase (NA), or both. Therefore, the composition of human influenza vaccines needs to be updated almost every year to match the newly circulating strains. Antigenic shift is caused by the substitution of HA and sometimes NA through gene reassortment, sometimes involving antigenically remote strains of animal origin (reviewed in [2]). Such an unpredictable event could cause pandemics, such as the 2009 H1N1 outbreak [2]. Since epidemics of the influenza A virus continue and the emergence of new highly lethal influenza viruses through animal-to-human transmission remains a constant threat, vaccines that can induce broadly protective immune responses against influenza A are urgently needed [2]. This problem can be solved by inducing cross-protective antibodies or T-cell responses against the conserved viral proteins, such as the conserved stalk domain of the HA, the matrix proteins M1 and M2, and the nucleoprotein [2,3].

The M2 protein, compared with other proteins encoded by the influenza A genome, is highly conserved [4,5]. M2 is a tetrameric membrane protein. Its extracellular domain (M2e) is a short (23 a.a.) peptide, the sequence of which is highly conserved in nearly all human isolates of the influenza A virus that circulated between 1918 and 2008 [2,5]. The use of M2e peptide would allow the development of a “universal” vaccine that could be efficient against a wide range of influenza strains. M2e is a poor immunogen and M2e-specific antibody responses are hardly induced following an infection [6]. However, anti-M2e immune responses could be enhanced by attaching M2e to a highly immunogenic carrier or adjuvant [5]. 

A most widely used approach to increase the immunogenicity of M2e is to obtain recombinant virus-like nanoparticles that display M2e on their surface [2]. Nanoparticle-based vaccines are highly immunogenic structures and can induce protective immunity [7,8,9,10,11,12]. The presentation of foreign epitopes on the surface of nanoparticles as a dense array of peptide antigens could induce a powerful immune response against them, even in the absence of an additional adjuvant [13]. Different studies demonstrated that M2e-based vaccines could be highly immunogenic and protect against infection [2,14,15]. Clinical studies have been conducted with M2e vaccine candidates, which demonstrated their safety and immunogenicity in humans [16]. However, M2e-based vaccines are still less effective than conventional hemagglutinin-based vaccines, so the search for optimal adjuvant carriers to present M2e in a highly immunogenic form continues [2].

In 2006, Raman et al. reported the design of a self-assembling peptide (SAP) that was able to oligomerize into icosahedral nanoparticles [17]. A 64 a.a. monomeric building block was composed of N-terminal 36 a.a. of the minor modified pentameric coiled-coil oligomerization helical domain of the cartilage oligomeric matrix protein [18], followed by 26 a.a. of de novo-designed trimeric coiled-coil domain [19]. These two oligomerization domains were joined by a linker consisting of two glycine residues. The cysteine residues in positions 33 and 42 were placed for the disulfide bridge formation between the helices. Self-assembly occurs when the coiled-coil domains of the monomers associate, forming a dodecahedral/icosahedral nanoparticle. The nanoparticle with regular geometry was composed of 60 monomeric building blocks [17]. SAP-based nanoparticles could be used as carriers of foreign epitopes. Such an approach ha been successfully used for the design of vaccine candidates for malaria [20,21], influenza A virus [22,23], SARS [24], HIV [25,26], toxoplasmosis [27,28], and bronchitis [29]. Particularly, SAP-based particles displaying M2e peptide and stalk HA epitopes of the influenza A virus induce potent humoral and T-cell responses and protect against the viral infection [30]. In these studies, recombinant SAP-based proteins were obtained in a bacterial expression system or in mammalian cells [26]. 

Recombinant proteins, including vaccines, can be produced in different expression systems, including bacteria, yeast, plants, and mammalian cells. The potential advantages of using plants are ease of scaling and high production volume [31,32,33,34,35,36]. Additionally, molecular farming in plants could facilitate the rapid production of the recombinant proteins at a large scale, as demanded in the case of a pandemic [37,38]. A high production level of the recombinant proteins in plants can be achieved through the use of transient expression systems based on agroinfiltration with recombinant plant viral vectors [39,40,41,42]. Plants have been used to produce vaccines, monoclonal antibodies, and other medically important recombinant proteins [37]. In 2022, the plant-made COVID-19 vaccine, Covifenz^®^, was first approved for use in Canada [43]. Such success has revived interest in the plant-based production of biopharmaceuticals for human use. 

In this study, we designed a fusion protein comprising four copies of M2e peptide and SAP. The protein was transiently expressed in *Nicotiana benthamiana* plants using a viral-based expression system and was found to form nanoparticles in vitro. The immunization of mice with the plant-produced SAP nanoparticles bearing M2e induced a strong humoral immune response and provided protective immunity against a lethal influenza A virus challenge.

## 2. Results

### 2.1. Composition of SAP-M2e Fusion Protein

**Figure 1:**
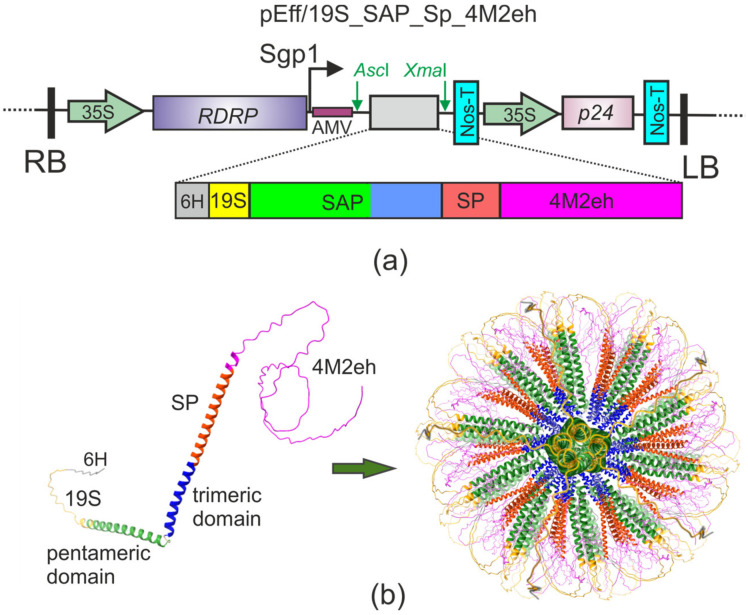
Expression vector and recombinant protein. Scheme of the expression vector (**a**). RDRP, RNA-dependent RNA polymerase gene; Sgp1, the first promoter of subgenomic RNA of PVX; AMV, translational enhancer from alfalfa mosaic virus; 35S, promoter of the cauliflower mosaic virus RNA; Nos-T, terminator of the *A. tumefaciens* nopaline synthase gene; P24, gene of suppressor of silencing from grapevine leafroll-associated virus-2. 6H, hexahistidine tag; 19S, flexible glycine-rich linker; SAP, self-assembling peptide; SP, rigid helical linker; 4M2eh, four tandem copies of M2e peptide; RB and LB, the right and left borders of T-DNA region. Models of the three-dimensional structure of monomeric 19S_SAP_Sp_4M2eh protein and nanoparticles composed of 60 monomers (**b**).

Four tandem copies of M2e peptide with human influenza A consensus sequence were used as the basis for the recombinant vaccine protein. The incorporation of multiple copies of M2e into the nanoparticle-forming protein should have increased the immune response against M2e, as previously shown for the HBc particles presenting this peptide [44]. In the M2e sequence, cysteines at positions 17 and 19 were replaced by serines to prevent the formation of disulfide bonds and protein aggregation; such modification does not affect M2e immunogenicity [45]. Four copies of M2e peptide were fused to the C terminus of SAP via a “hard” 28-amino-acid-long alpha-helical linker (SP) to facilitate the appropriate folding of the recombinant proteins [46]. The N-terminal hexahistidine tag was separated from the protein by a 19 a.a. long flexible glycine-serine linker (19S) to facilitate protein purification using nickel-affinity chromatography (Figure 1a).

Computational modeling was used to predict the spatial structure of 19S_SAP_Sp_4M2eh. The structure of a single monomeric peptide building block was predicted using Alphafold v2.3.1 [47]. The structure of nanoparticles composed of 60 units was reconstructed according to icosahedron symmetry. The obtained structure predicted that M2e peptides are exposed on the surface of the particles (Figure 1b).

### 2.2. Expression and Purification of Recombinant 19S_SAP_Sp_4M2eh Protein

**Figure 2:**
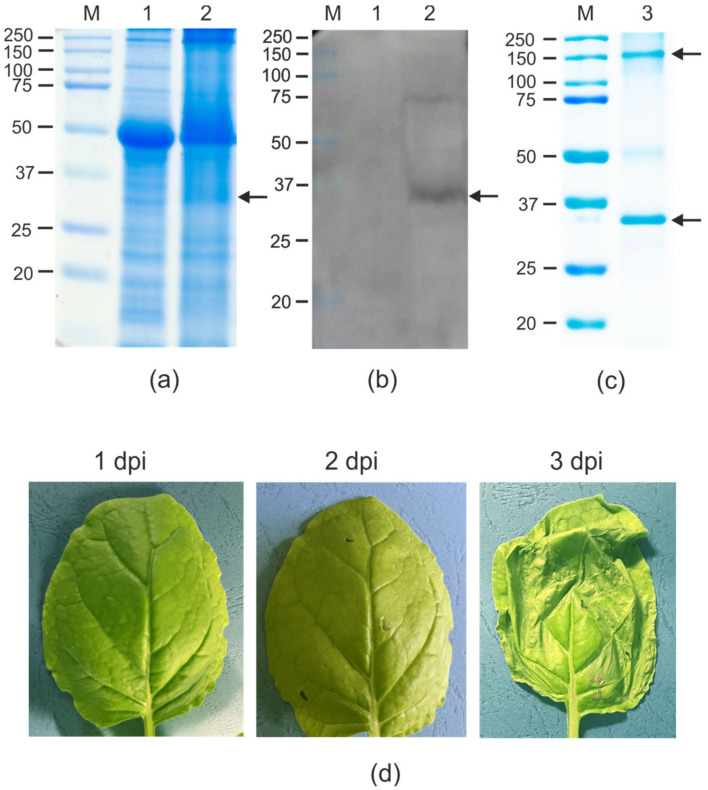
Expression of 19S_SAP_Sp_4M2eh protein in *N. benthamiana* plants. Coomassie brilliant blue-stained gel (**a**,**c**) and Western blotting with antibodies against M2e (**b**) of proteins isolated from plants. M, molecular weight marker (kD); Lanes: 1, total proteins isolated from non-infiltrated leaf; 2, total proteins isolated from leaf infiltrated with pEff/19S_SAP_Sp_4M2eh; 3, purified 19S_SAP_Sp_4M2eh protein. Position of 19S_SAP_Sp_4M2eh protein is shown by arrow. Photographs of *N. benthamiana* leaves infiltrated with pEff/19S_SAP_Sp_4M2eh on different dpi (**d**).

The 19S_SAP_Sp_4M2eh protein was expressed in *N. benthamiana* plants using the pEff viral vector [42] (Figure 1a). *N. benthamiana* leaves were infiltrated with agrobacteria carrying the recombinant vector pEff/19S_SAP_Sp_4M2eh. Necrosis in the infiltration zones was observed already 2–3 days post-infiltration (dpi) (Figure 2d). Probably, this effect is associated with the toxicity of SAP for plant cells, since the expression of SAP without M2e using the pEff vector caused necrosis already on the second day after infiltration.

The protein samples isolated from the agroinfiltrated leaves were analyzed using SDS-PAGE (Figure 2a) and Western blotting (Figure 2b). Western blotting with antibodies against M2e showed that 19S_SAP_Sp_4M2eh was efficiently expressed in plant cells at the level of about 80–100 μg/g of fresh leaf tissue. Because the fusion protein appeared to be completely insoluble, its purification using metal affinity chromatography was performed under denaturing conditions. 

After purification on the Ni-NTA sorbent, the protein sample still contained a large fraction of plant RuBisCo (ribulose-1,5-bisphosphate carboxylase/oxygenase). Therefore, the obtained protein was re-purified using the same protocol.

### 2.3. Protein Refolding and In Vitro Assembly of Nanoparticles

To carry out protein renaturation and its self-assembly into nanosized particles in vitro, the obtained protein sample was subjected to stepwise dialysis against PBS with decreasing concentrations of urea. As a result, a homogeneous soluble protein sample with a minimal content of RuBisCo was obtained (Figure 2c). The final yield of purified fusion protein was about 60 μg/g of fresh leaf tissue.

The calculated molecular weight of the 19S_SAP_Sp_4M2eh protein is 25 kD, however, it migrated slowly in SDS-PAGE (~33–34 kD, Figure 2). Probably, the abnormal mobility of this protein in SDS-PAGE is due to a large proportion of negatively charged amino acid residues (15 Asp and 32 Glu of 231 a.a.) [48]. Along with the presumably monomeric form of the protein, SDS-PAGE also revealed multimers with an apparent molecular weight of about 150 kDa, which may represent SAP pentamers.

**Figure 3:**
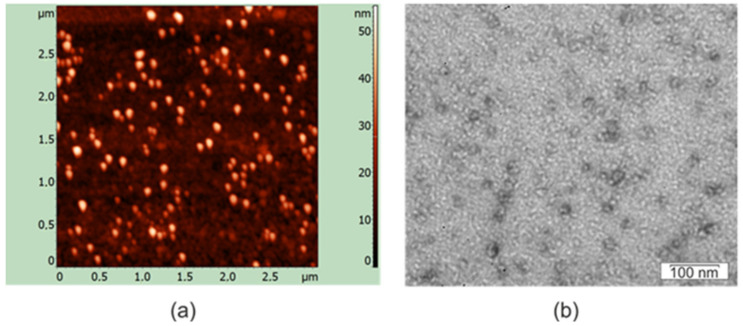
Analysis of virus-like particles formed by 19S_SAP_Sp_4M2eh protein by atomic force microscopy (**a**) and transmission electron microscopy (**b**).

The assembly of the 19S_SAP_Sp_4M2eh protein into nanosized structures was analyzed by electron and atomic force microscopy. Spherical particulate structures were observed by both methods (Figure 3). According to transmission electron microcopy, the particles had a diameter of about 15–25 nm. The size of 19S_SAP_Sp_4M2eh particles estimated by atomic force microscopy was about 15–40 nm. Particle size distribution histograms are shown in Supplemental Figure S1.

### 2.4. Immunogenicity and Protectivity of Plant-Produced 19S_SAP_Sp_4M2eh

**Figure 4:**
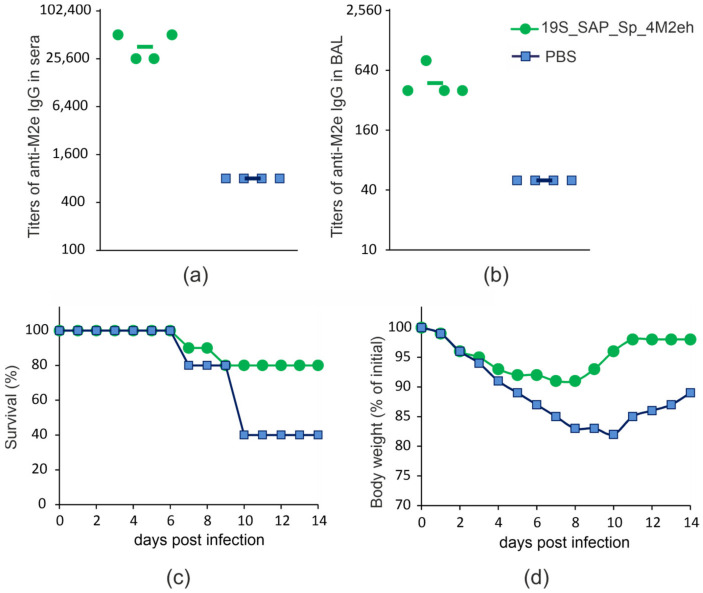
Immunogenicity and protective efficiency of 19S_SAP_Sp_4M2eh nanoparticles. Titers of IgG antibodies to synthetic M2e peptide in sera (**a**) and BAL (**b**) of immunized mice after the third immunization. The values for 4 animals (circles and squares) and the geometric mean titers (horizontal lines) are shown. The survival rate (**c**) and body weight changes (**d**) of immunized and control (PBS) mice was monitored for 14 days post-challenge with 4 × LD_50_ of A/Aichi/2/68 (H3N2) influenza A virus.

To characterize the immunogenicity and protective action of the candidate vaccine, mice were immunized subcutaneously with purified nanoparticles formed by the 19S_SAP_Sp_4M2eh protein. The sera and broncho-alveolar lavage (BAL) fluids were taken after the third immunization and analyzed by ELISA to identify IgG antibodies directed against M2e. A strong M2e-specific immune response developed in immunized mice (Figure 4a,b). The titers of anti-M2e IgG in sera and mucosal secretions were significantly higher than in the control group receiving PBS (*p* < 0.001 in sera and *p* < 0.05 in BAL).

To evaluate the protective action of the 19S_SAP_Sp_4M2eh protein, the immunized and the control mice were challenged with the mouse-adapted human influenza strain A/Aichi/2/68 (H3N2). As shown in Figure 4c, 80% of the immunized mice survived the challenge with 4xLD_50_, while the rate of survival among the control group was 40%.

The morbidity of the disease was monitored by measuring the weight of the mice. The data presented in Figure 4 showed that immunization did not completely eliminate morbidity, but strongly reduced it relative to the control. The maximum weight loss in the immunized group was 9%, while the surviving mice in the control group lost up to 17% of weight and recovered more slowly (Figure 4d).

## 3. Discussion

One of the actively developing areas in immunology is the development of vaccines based on nanosized virus-like particles that significantly increase the immunogenicity of the peptides exposed on their surface. Capsid proteins of viruses, which can spontaneously assemble into nanosized structures, are used to obtain VLPs most often. The insertion of foreign epitopes into capsid proteins allows for the obtaining of recombinant capsids which assemble into VLPs, presenting a dense array of epitopes on the surface, which is known to be optimal for a vigorous immune response. Although rather large proteins (for example, GFP) could be presented on the surface of VLPs formed by viral capsids, such insertions often change the spatial structure of the capsid protein, preventing the assembly of particles. As an alternative building block for the creation of VLP, artificial peptides capable of self-aggregation can be used. An example is a 63 a.a. long SAP peptide, which is formed by nanoparticles with regular dodecahedral symmetry composed of 60 monomeric peptide chains. SAP was successfully used for the presentation of various short antigens [20,21,22,23,24,25,26,27,28,29].

In a previous work, we used SAP as a carrier for the presentation of the M2e peptide of the influenza virus on the surface of such artificial nanoparticles. The recombinant protein contained four tandem copies of the M2e peptide attached to the SAP peptide at its C-terminus. The protein was successfully expressed in *E. coli*, and upon refolding in vitro, it self-assembled into spherical nanoparticles with a size of 30–50 nm [30].

The purpose of this work was the development of a plant-produced candidate influenza vaccine based on SAP particles bearing M2e peptides. A transient expression system as an alternative to the creation of transgenic plants is fast and simple. This method simplifies the creation of gene expression cassettes and allows the expression of recombinant proteins to be scaled up quickly and easily [49,50,51]. Plant viral vectors enable the production of recombinant proteins at a high level (up to 5 mg per gram of plant biomass) within several days [52,53]. 

For the expression of the SAP-M2e recombinant protein in *N. bethamiana* plants, we used the self-replicating viral vector pEff genome [42] that was used for the production of different recombinant proteins [40,54,55,56]. The 19S_SAP_Sp_4M2eh protein was successfully expressed at the level of about 100 μg/g of fresh leaf biomass, and the purification yield was about 60 μg per gram. However, the yield of 19S_SAP_Sp_4M2eh was about ten times lower than the previously reported maximum level for the pEff expression system [42]. Some M2e fusions were also expressed in plants using the pEff system at higher levels, including up to 1 mg/g for flagellin-M2e [57], and 300–400 μg/g for M2e fused to the Hepatitis E capsid protein [58]. Nevertheless, the expression level of 19S_SAP_Sp_4M2eh was several times higher than those achieved using a similar expression system for the production of M2e-bearing VLPs based on the core antigen of the hepatitis B virus in plants [59].

Therefore, it is likely that the inclusion of the SAP peptide in the fusion protein resulted in lower expression. The SAP peptide is highly hydrophobic and probably toxic to the plant cells, as indicated by the appearance of the necrosis of plant tissue already two days after agroinfiltration, while the maximal levels of expression with pEff-like vectors are usually achieved only on the fourth or fifth day [42].

The purification of the plant-produced target protein under denaturing conditions using metal affinity chromatography and subsequent stepwise dialysis resulted in the hybrid protein self-assembling in vitro into spherical nanoparticles with a size of about 15–30 nm. The empty SAP peptide after a similar refolding procedure formed nanoparticles that were about 16 nm in diameter [17]. Probably, the attachment of four copies of M2e peptide to SAP led to an increase in particle size, consistently with the predicted localization of M2e on the surface of recombinant particles (Figure 1).

Recombinant 19S_SAP_Sp_4M2eh nanoparticles were administered subcutaneously to Balb/c mice 3 times with an interval of 2 weeks at a dose of 50 μg. Immunization induced high levels of anti-M2e antibodies both in sera and BAL and provided 80% protection against the A/Aichi/2/68 (H3N2) influenza virus. Similar survival rates (90%) were reported for mice that were immunized with 19s_SAP_Sp_4M2eh produced in *E. coli* and challenged with A/PR/8/34 (H1N1) or A/Aichi/2/68 (H3N2) viruses [30]. Therefore, M2e-bearing nanoparticles obtained in bacterial and plant expression systems have similar immunogenic characteristics.

Overall, the obtained SAP nanoparticles carrying the M2e peptide of the influenza A virus are a good basis for the development of a plant-produced influenza vaccine.

## 4. Materials and Methods

### 4.1. Structure of the SAP-Based Fusion Protein

The following peptides were included in the fusion protein: SAP (DME LRE LQE TLA ALQ DVR ELL RQQ VKQ ITF LKC LLM GGR LLC RLE ELE RRL EEL ERR LEE LER R)—self-assembling peptide, modified positions compared to previously described [17] are underlined; Sp (EAAAKEAAAKEAAAKEAAAKEAAAK)—rigid helical linker [46]; 19S (GTSGSSGSGSGGSGSGGGG)—flexible glycine rich linker [60]; and M2eh (SLLTEVETPIRNEWGSRSNDSSD)—consensus sequence of the M2e of the human influenza A virus strains with the replacement of cysteines by serines [45]. The fusion protein was arranged as 19S-SAP-Sp-4M2eh.

### 4.2. Expression Vector

The potato virus X (PVX)-based vector pEff [42] was used for the expression of the recombinant 19S_SAP_Sp_4M2eh protein. This vector contains the 5′-untranslated region of the PVX genome, the gene for RNA-dependent RNA polymerase enabling the replication of the vector in a plant cell, the promoter of the first viral subgenomic RNA, the AMV translation enhancer (5′-nontranslated region of RNA 4 of the alfalfa mosaic virus), the *gfp* gene flanked by unique *Asc*I and *Sma*I sites that can be replaced by the gene of the interest, and the 3′-untranslated fragment of the PVX genome. pEff also contains an expression cassette for the P24 suppressor of silencing from grapevine leafroll-associated virus-2. Both cassettes are located between the 35S promoter of the cauliflower mosaic virus and the Nos-T terminator of the nopaline synthase gene from *Agrobacterium tumefaciens*. All these genetic elements are located in the T-DNA region of a binary vector that could be maintained in both *E. coli* and *A. tumefaciens*. This pEff vector was previously used for the fast high-level expression of recombinant proteins in plant cells [40,55,56,59].

The sequence encoding 19S_SAP_Sp_4M2eh with the N-terminal hexahistidine tag was amplified by PCR using primers AscI-Pr-F PalI his (ata tGG CGC GCC ATG AGA GGA TCG CAT CAC) and Pr-R M2eh-BlpI_SmaI (ata tCC CGG GTC CAA GCT CAG CTA ATT AAG) and plasmid pQE30/19s_SAP_Sp_4M2eh as a template [30]. The PCR fragment was cloned in pEff at the *Asc*I/*Sma*I sites, resulting in the expression vector pEff/19S_SAP_Sp_4M2eh. Upon construction in *E. coli*, this vector was transferred into the *A. tumefaciens* strain GV3101 via electroporation. Agrobacteria were grown in LB at 28 °C with kanamycin (50 μg/mL), rifampicin (50 μg/mL), and gentamycin (25 μg/mL).

### 4.3. Agroinfiltration of N. benthamiana Leaves

An agrobacterium-mediated system was used for transient expression in *N. benthamiana* leaves [61]. Plants were grown in a greenhouse under a 16 h daylight regime with additional illumination with full spectrum phyto-lamps. The recombinant *A. tumefaciens* GV3101 strain carrying the pEff/19S_SAP_Sp_4M2eh vector was grown overnight in a shaking incubator at a temperature of 28 °C. The *A. tumefaciens* cells were pelleted by centrifugation for 5 min at 4000× *g*, and resuspended in 10 mM MES (pH 5.5) and 10 mM MgSO_4_ to an OD_600_ of 0.2. Bacterial cells were infiltrated into plant cells using a syringe without a needle. Leaves were harvested 2–3 days after infiltration (dpi).

### 4.4. SDS-PAGE and Western-Blotting of the Proteins Isolated from Plants

For small-scale expression, pieces of infiltrated leaves (8–10 mg) were excised and homogenized in 50 μL of the PBS buffer (50 mM sodium phosphate pH 7.2, 300 mM NaCl). The resulting suspension was mixed with half volume of the 3x sample loading buffer (40% glycerol, 4% SDS, 50 mM Tris pH 6.8, 1% bromophenol blue, 5% beta-mercaptoethanol), resulting in a total protein fraction for SDS-PAGE. Then, 10 µL of the obtained mixture (corresponding to about 1 mg of leaf tissue) was analyzed by SDS-PAGE and Western blotting. After electrophoresis, the gel was stained with One-Step Blue Protein Gel Stain (BIOTIUM, San Francisco, CA, USA) or was used for protein transfer onto a Hybond-P membrane (GE Healthcare, Chicago, IL, USA) by semi-dry transfer using the Trans-Blot Turbo Transfer System (Bio-Rad Laboratories, Hercules, CA, USA). Afterwards, the membrane was blocked with a 5% (*w/v*) solution of dry milk in TBS-T (20 mM Tris pH 8.0, 150 mM NaCI, 0.1% Tween 20) buffer for 1 h at room temperature and subsequently incubated with mouse polyclonal anti-M2e primary antibodies (used at a dilution of 1:30,000) for 1 h at room temperature. The membrane was washed 3 times with TBS-T buffer (15 min at room temperature) and incubated with secondary rabbit anti-mouse antibodies conjugated with peroxidase (Promega, Madison, WI, USA) for 1 h at room temperature. Then, the membrane was washed 3 times with TBS-T buffer (15 min at room temperature). Specific protein–antibody complexes were visualized using a Western Blot ECL Plus kit (GE Healthcare, New York, NY, USA) and chemiluminescence detector Fusion Solo X (Vilber, Eberhardzell, Germany).

### 4.5. Isolation and Purification of Recombinant Proteins from Plant Biomass

For large-scale isolation, the plant-produced 19S_SAP_Sp_4M2eh protein was purified on Ni-NTA resin (QIAGEN, Hilden, Germany) under denaturing conditions. Two to three days after infiltration, the *N. benthamiana* leaves were harvested and homogenized in a solution containing 6 M guanidine-HCI, 50 mM phosphate-buffered saline (PBS), and 300 mM NaCI (pH 7.2). For complete protein denaturation, incubation in the buffer was carried out for 12 h at +10 °C. The obtained mixture was then subjected to centrifugation (14,000× *g* for 15 min). The supernatant was loaded onto Ni-NTA resin pre-incubated with the same buffer and allowed to stand for 60 min. The resin was subsequently washed with the buffers (I) 6 M guanidine-HCI, 50 mM PBS, 300 mM NaCI, and 5 mM imidazole; and (II) 6 M urea, 50 mM PBS, 300 mM NaCI, and 10 mM imidazole. The recombinant protein 19S_SAP_Sp_4M2eh was eluted from the resin in a buffer with 6 M urea, 50 mM PBS, 300 mM NaCI, and 500 mM imidazole. 

After elution, the protein was dialyzed against the buffer containing 6 M urea, 50 mM PBS, and 300 mM NaCI. The recombinant protein was then purified again on Ni-NTA resin as described above, but the concentration of imidazole in the first and second washes was increased to 16 and 20 mM, respectively. After elution, the protein was step-by-step dialyzed against PBS buffers (50 mM PBS, 300 mM NaCI) containing 4 M urea, 2 M urea, 1 M urea, 0.5, and finally without urea.

### 4.6. Structural Analysis of Nanoparticles

The particles formed by the recombinant protein after refolding were examined using a transmission electron microscope and atomic force microscope. Electron microscopy was performed on a JEM 1400 instrument (JEOL, Tokyo, Japan). The purified proteins were placed on carbon-formvar-coated copper grids (TED PELLA, Redding, CA, USA) and stained with 1% (*w/v*) uranyl acetate in methanol. Particle sizes (*n* = 20) in digital photographs were determined using the ImageJ software [62]. 

Atomic force microscopy was performed using an Integra Prima microscope and Nova SPM software (NT-MDT, Moscow, Russia). The scanning was performed in the semi-contact mode using gold cantilever NSG01 (NT-MDT). Particle sizes (*n* = 20) were determined using the NT-MDT Nova v. 1.06.26 software supplied with the instrument.

### 4.7. Mouse Immunization

Female BALB/c mice (16–18 g) (*n* = 14) were immunized subcutaneously 3 times at 2-week intervals with 50 µg of protein with a sodium desoxyribonucleate (Derinat, 1000 µg) as an adjuvant. Derinat is licensed for human use in the Russian Federation as an immunomodulator). The control group was subcutaneously injected with PBS (*n* = 14). The study was carried out in accordance with the Russian Guidelines for the Care and Use of Laboratory Animals, and the protocol was approved by the Committee for Ethics of Animal Experimentation at the Research Institute of Influenza.

### 4.8. Antibody Detection by ELISA

Sera samples from sera and broncho-alveolar lavages (BALs) of four mice were isolated after the second and third immunization. Antigen-specific levels of antibodies in serum and BAL were determined by ELISA in 96-well microtiter plates (Greiner, Germany) coated overnight at 4 °C with the synthetic peptide M2eh (SLLTEVET PIRNEWGCRCNDSSD, 5 µg/mL) in PBS (pH 7.2). As a conjugate, goat polyclonal anti-mouse IgG (Abcam, UK) labeled with a horseradish peroxidase was used. After adding tetramethylbenzidine substrate (Biolegend, San Diego, CA, USA) and monitoring color development, the reaction was stopped by H_2_SO_4_, and OD at 450 nm was measured on a microplate spectrophotometer.

### 4.9. Influenza Virus and Challenge

Mouse-adapted A/Aichi/2/68 (H3N2) influenza A virus obtained from the Collection of Influenza and Acute Respiratory Viruses at the Research Institute of Influenza was used to challenge the immunized mice (ten in each group) at a dose of 4 × LD_50_. Two weeks after the third immunization, the virus was administered intranasally in a total volume of 50 µL to the mice anaesthetized by ether. The mice from each group were monitored daily for the survival rate following the viral challenge and mass changes for a period of 2 weeks. Experimental work with influenza strains was carried out in a BSL2 facility.

### 4.10. Ethics Statement

The study was carried out according to the Russian Guidelines for the Care and Use of Laboratory Animals. The protocol was approved by the Committee for Ethics of Animal Experimentation at the Research Institute of Influenza (Permit ID 13/a, approved 21 October 2019). All possible efforts were made to minimize the suffering of the animals.

## 5. Conclusions

A recombinant fusion protein comprising the artificial self-assembling peptide SAP and four tandem copies of the influenza A virus M2e peptide can be transiently expressed in plants using the pEff viral vector at a level of about 80–100 μg/g of fresh leaf tissue. The plant-produced recombinant protein was purified using metal affinity chromatography under denaturing conditions. After renaturation, it was capable of self-assembling in vitro into spherical nanoparticles 15–30 nm in size. The immunization of mice with recombinant nanoparticles induced a strong M2e-specific immune response in sera and mucosal secretions, and protected animals that were challenged with the influenza A virus. SAP-based nanoparticles carrying M2e peptides can be further used to develop a recombinant “universal” vaccine against influenza A produced in plants.

## Data Availability

Not applicable.

## Supplementary Materials

The following supporting information can be downloaded at: https://www.mdpi.com/article/10.3390/plants12112228/s1, Figure S1: Particle size distribution histograms determined from atomic force microscopy and transmission electron microscopy images.
