# Supplementary material for: Plant-Produced Nanoparticles Based on Artificial Self-Assembling Peptide Bearing the Influenza M2e Epitope
Source: Plants (Basel). 2023 Jun 5;12(11):2228. doi: 10.3390/plants12112228 (PMC10255905; doi:10.3390/plants12112228)
Supplement: Supplementary file 1 [file plants-12-02228-s001.zip › Figure S1.pdf]

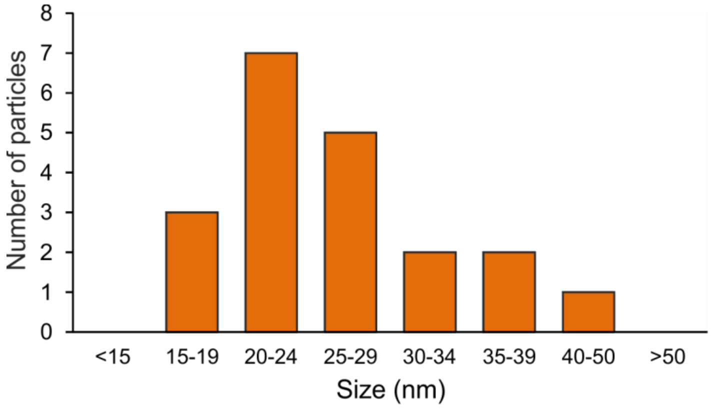


(a)

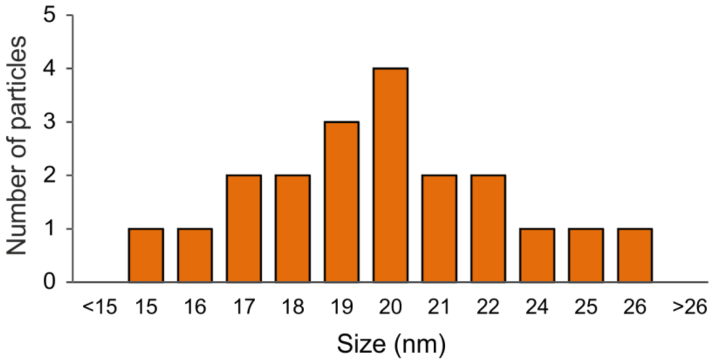


(b)

**Supplemental Figure S1.** Particle size distribution histograms determined from the atomic force microscopy (a) and transmission electron microscopy (b) images.

Sizes of 20 particles were measured in each experiment. The following particle sizes were obtained:
Atomic force microscopy: 16 nm (1), 17 nm (1), 18 nm (1), 20 nm (3), 21 nm (1), 22 nm (1), 23 nm (1), 24 nm (1), 25 nm (2), 26 nm (1), 27 nm (1), 28 nm (1), 30 nm (1), 31 nm (1); 35 nm (1), 38 nm (1), 50 nm (1).
Transmission electron microscopy: 15 nm (1), 16 nm (1), 17 nm (2), 18 nm (2), 19 nm (3), 20 nm (4), 21 nm (2), 22 nm (2), 24 nm (1), 25 nm (1), 26 nm (1).
Numbers in parentheses indicate the number of analyzed particles of a given size.
